# Australian guideline on wound healing interventions to enhance healing of foot ulcers: part of the 2021 Australian evidence-based guidelines for diabetes-related foot disease

**DOI:** 10.1186/s13047-022-00544-5

**Published:** 2022-05-25

**Authors:** Pamela Chen, Keryln Carville, Terry Swanson, Peter A. Lazzarini, James Charles, Jane Cheney, Jenny Prentice, on behalf of the Australian Diabetes-related Foot Disease Guidelines & Pathways Project, Stephen Twigg, Peter Lazzarini, Anita Raspovic, Jenny Prentice, Robert Commons, Robert Fitridge, James Charles, Jane Cheney

**Affiliations:** 1 School of Medicine, University of Tasmania, Hobart, Australia; 2 Podiatric Medicine and Surgery, School of Allied Health, The University of Western Australia, Perth, Australia; 3 Joondalup Health Campus, Ramsay Healthcare Australia, Perth, Australia; 4 Silver Chain Group and Curtin University, Perth, Australia; 5 Nurse Practitioner, Warrnambool, Victoria Australia; 6 School of Public Health and Social Work, Queensland University of Technology, Brisbane, Australia; 7 Allied Health Research Collaborative, The Prince Charles Hospital, Brisbane, Australia; 8 First Peoples Health Unit, Faculty of Health, Griffith University, Gold Coast, Queensland Australia; 9 Diabetes Victoria, Melbourne, Australia; 10 Hall and Prior Health and Aged Care Group, Perth, Australia; 11 Diabetes Feet Australia, Brisbane, Australia; 12 Australian Diabetes Society, Sydney, Australia

**Keywords:** Diabetes-related foot ulcer, Diabetic foot, Foot ulcer, guideline, Recommendations, Treatment, Wound healing, Wound treatment

## Abstract

**Background:**

Diabetes-related foot ulceration (DFU) has a substantial burden on both individuals and healthcare systems both globally and in Australia. There is a pressing need for updated guidelines on wound healing interventions to improve outcomes for people living with DFU. A national expert panel was convened to develop new Australian evidence-based guidelines on wound healing interventions for people with DFU by adapting suitable international guidelines to the Australian context.

**Methods:**

The panel followed National Health and Medical Research Council (NHMRC) procedures to adapt suitable international guidelines by the International Working Group of the Diabetic Foot (IWGDF) to the Australian context. The panel systematically screened, assessed and judged all IWGDF wound healing recommendations using ADAPTE and GRADE frameworks for adapting guidelines to decide which recommendations should be adopted, adapted or excluded in the Australian context. Each recommendation had their wording, quality of evidence, and strength of recommendation re-evaluated, plus rationale, justifications and implementation considerations provided for the Australian context. This guideline underwent public consultation, further revision and approval by ten national peak bodies.

**Results:**

Thirteen IWGDF wound healing recommendations were evaluated in this process. After screening, nine recommendations were adopted and four were adapted after full assessment. Two recommendations had their strength of recommendations downgraded, one intervention was not currently approved for use in Australia, one intervention specified the need to obtain informed consent to be acceptable in Australia, and another was reworded to clarify best standard of care. Overall, five wound healing interventions have been recommended as having the evidence-based potential to improve wound healing in specific types of DFU when used in conjunction with other best standards of DFU care, including sucrose-octasulfate impregnated dressing, systemic hyperbaric oxygen therapy, negative pressure wound therapy, placental-derived products, and the autologous combined leucocyte, platelet and fibrin dressing. The six new guidelines and the full protocol can be found at: https://diabetesfeetaustralia.org/new-guidelines/

**Conclusions:**

The IWGDF guideline for wound healing interventions has been adapted to suit the Australian context, and in particular for geographically remote and Aboriginal and Torres Strait Islander people. This new national wound healing guideline, endorsed by ten national peak bodies, also highlights important considerations for implementation, monitoring, and future research priorities in Australia.

**Supplementary Information:**

The online version contains supplementary material available at 10.1186/s13047-022-00544-5.

## Background

Diabetes-related foot disease causing diabetes-related foot ulceration (DFU) is one of the most devastating complications of diabetes, precedes up to 75% of amputations in people with diabetes and accounts for a significant proportion of the global disability burden [1, 2]. Australia has a national prevalence of diabetes of 5.3% [3] and amongst those living with diabetes, the prevalence of diabetes-related foot ulceration is around 3.2% [4]. DFUs cost the healthcare systems an estimated $1.6 billion [5, 6], affect around 50,000 people, cause 28,000 hospital admissions and 5000 amputations each year [5, 6]. A further 300,000 Australians are living with diabetes-related peripheral neuropathy and thus at risk of DFU. Aboriginal and Torres Strait Islander people are disproportionately affected by DFU, being up to 38 times more likely to have a major amputation compared to their non-Indigenous counterparts [6–8]. It is thus critical that interventions to enhance or facilitate healing of DFU are supported by strong evidence of benefit and cost-effectiveness [9], and all communities across Australia should have equitable access to these interventions [8].

Management of DFU has been a global priority with new International Working Group of the Diabetic Foot (IWGDF) Guidelines released every 4 years since 1999 and the most recent released in 2019 [9]. In contrast, the last national evidence-based guideline in Australia was released in 2011 [10], and is now considerably outdated [5, 11]. Furthermore, in 2011, the IWGDF Guidelines reported that poor study design and reporting were a key limitation in the evidence base of interventions to enhance healing of DFU [12, 13]. However, in 2016 with the subsequent IWGDF publication of international best practice reporting standards for DFU studies [14], several well-designed clinical trials involving interventions to enhance wound healing in DFU have since been published [15, 16].

With this in mind, not only did the Australian Guideline (2011) urgently need updating, but experts suggested there was a ‘renaissance in DFU wound healing intervention studies’ [17] that provided considerable new robust evidence to support different wound healing interventions with significantly improved outcomes for people with DFU. Thus, this is a timely opportunity to adapt the 2019 IWGDF Guideline to the Australian context to become the new Australian guideline on wound healing interventions to heal DFU which should help guide health professionals, address the large national DFU burden and mitigate existing inequalities amongst Australians living with DFU.

## Methods

The methods to develop this guideline have been described in detail in the accompanying guidelines development protocol authored by the Australian Diabetes-related Foot Disease (DFD) Guideline Development Working Group [18]. Eight overarching steps recommended by the National Health and Medical Research Council (NHMRC) for adapting source guidelines [18–20] were followed and they were: 1) defining the scope, 2) identifying potential source guidelines, 3) assessing the suitability of source guidelines, 4) assessing and deciding which source guideline recommendations to adopt, adapt or exclude, 5) drafting new recommendations and rationale for the context, 6) collating recommendations and rationales into new guidelines, 7) developing clinical pathway(s) to aide implementation, and 8) consultation and endorsement of the final guideline [20]. Steps 1–3 were performed by the Guideline Development Working Group [18] who systematically searched for and identified all available potential source guidelines, and assessed the 2019 IWGDF Guideline [9] as the only suitable international sourced guideline to adapt to the Australian context. The remaining methodological steps are the subject of this paper and are described below.

### National panel

The authors (‘the panel’) of this Australian wound healing interventions guideline are all national experts of a national expert panel invited by the Australian DFD Guidelines Development Working Group to systematically adapt the IWGDF recommendations on wound healing interventions to the Australian context. Each panel member was invited based on their recognised inter-disciplinary expertise in wound healing for people with DFU, along with a consumer, and an Aboriginal Torres Strait Islander DFU representative [18]. The panel was provided with the IWGDF Guideline (2019), systematic review [21] and supplementary documentation for wound healing interventions to screen, assess and decide on the applicability and acceptability of all 13 IWGDF Guideline (2019) wound healing intervention recommendations to the Australian context [9, 21].

### Screening recommendations

Each of the thirteen IWGDF (2019) recommendations were initially independently screened by two panel members to assess the quality of evidence, strength of recommendation, acceptability and applicability in the Australian context using a customised 7-item ADAPTE evaluation format [18, 22]. Any disagreement amongst the two panel members was discussed until consensus was reached and if this was not possible a third panel member was invited to aid decision making. The full panel subsequently met to discuss and reach consensus on all item ratings for all recommendations. Each recommendation where the panel agreed unanimously with the IWGDF on all quality of evidence and strength of recommendation item ratings, as well as deeming the recommendation applicable and acceptable to the Australian context, were adopted for use. Any recommendations where the panel were unsure if they agreed with the IWGDF on any of those items were referred for a full assessment at the next stage [18].

### Assessing recommendations

The GRADE Evidence to Decision (EtD) tool template was used for recommendations necessitating a full assessment [18, 19, 23, 24]. The EtD template facilitates judgements to be made on eight criteria: the problem, desirable effects, undesirable effects, quality (or certainty) of evidence, values (of importance of outcomes), balance of effects, acceptability and feasibility [19, 23, 24]. One panel member extracted all relevant evidence that supported the recommendation concerned from the IWGDF Guideline (2019) and/or systematic review to populate the EtD tool [9, 21]. The member then added any Australian literature or expert opinion considerations that were not included in the IWGDF Guideline (2019) that they considered necessary. Once populated, the EtD tool was checked by a second panel member and disagreements were discussed and resolved until consensus was achieved. All detailed and summary judgement items for each criterion were then rated in the populated EtD tool by a panel member and checked by another, and disagreements were once again discussed until a majority consensus was achieved. At this stage, the full panel met again to discuss and achieve consensus on the summary judgement ratings for all eight EtD criteria and then compared those judgements with those of the IWGDF Guideline (2019, 23, 24).

### Decisions on recommendations

The decision to adopt, adapt or exclude each original IWGDF Guideline (2019) recommendation was based on the level of agreement between the panel and the IWGDF judgements [23, 24]. If there was agreement between the panel and IWGDF across all or the vast majority of the eight criteria the recommendation was adopted. If there was disagreement, the recommendation could be adapted and if there were substantial disagreement and/or the panel concluded the acceptability or applicability was not adequate for the Australian context, it could be excluded [23, 24]. Where consensus was not achieved amongst the panel, the Australian DFD Guideline Development Working Group were consulted and assisted with this process if required.

Recommendations adapted at this stage of the process were re-evaluated on their quality of evidence and strength of recommendation until consensus, based on the panel’s summary judgements of the eight EtD criteria [23, 24]. GRADE defines quality of evidence as: high, if the panel were confident the findings from the supporting evidence were from studies with low risk of bias with reported consistent effects and further research was unlikely to change that confidence; moderate, if the panel had moderate confidence in the findings being of low risk of bias and/or with consistency of effects and further research was likely to impact that further; low, if the panel had low confidence in the findings being of risk of bias and with consistent effects; and very low, if there was very low confidence in the supporting evidence [23, 24]. The strength of recommendation was made taking into consideration the balance of effects, quality of evidence, values, applicability and acceptability in the Australian context [23, 24]. In brief, it was rated as strong, if there was a moderate-to-large difference in the balance of effects between the intervention and control; and weak, if there was a mild-to-moderate difference or any major uncertainty [23, 24]. The redrafted wording for adapted recommendations were based on the original IWGDF Guideline (2019) recommendations wording and adapted to reflect the Australian context in line with the panel’s specific judgement and the GRADE system.

### Drafting recommendations

Lastly, the panel drafted clear rationales for final decisions, summary justifications for the recommendations and detailed justifications on each EtD criteria if the recommendation was fully assessed. The panel also specified considerations for implementation of each recommendation in Australia as well as applicability to special subgroups, including geographically remote individuals and Aboriginal and Torres Strait Islander populations. Final considerations were given to monitoring of outcomes and potential future research priorities [23, 24]. The wound healing intervention guideline manuscript was drafted for inclusion as part of the Australian DFD Guideline (2021) and in preparation for public consultation [18].

### Consultation and endorsement

The draft guideline manuscript underwent a four-week public consultation period where all interested health professionals and peak national bodies were able to provide feedback on the draft via the completion of a 23-item customised consultation survey. The survey was designed based on ADAPTE examples with additional open ended items for feedback on each recommendation and overall final comments [18, 22]. At the completion of the four-week period, all consultation survey feedback was collated, analysed and reviewed to determine any necessary revisions to the guideline. Finally, the authors sought endorsement from the Australian DFD Guidelines Development Working Group and other relevant peak national bodies. The results of this paper detailed below contain all final recommendations in the new Australian guideline on wound healing interventions to enhance healing of foot ulcers.

## Results

After screening 13 IWGDF wound healing-related recommendations, nine were adopted and four required full assessment (see Table 1). Of the four recommendations undergoing full assessment, all were adapted for the following reasons: two recommendations had their strength of recommendation downgraded, one intervention was not currently approved for use in Australia, one intervention specified the need to obtain informed consent to be acceptable in Australia, and another was reworded to clarify best standard of care (see Table 2). The exact wording differences between the IWGDF Guideline (2019) and the new Australian Guideline (2021) recommendations are summarised in Table 3.

**Table 1:** Summary of screening ratings for acceptability and applicability in the Australian context for all IWGDF wound healing recommendations

Recommendation	Acceptability			Applicability				Full assessment	Comments
	1	2	3	4	5	6	7		
1	+	+	+	+	+	+	+	Not required	
2	+	+	+	+	+	+	+	Not required	
3	+	+	+	+	+	+	+	Not required	
4	+	+	+	+	+	+	+	Not required	
5	+	+	+	+	+	+	+	Not required	
6	+	+	+	+	+	+	+	Not required	
7	+	+	+	+	+	+	+	Not required	
8	+	+	+	+	+	+	+	Not required	
9	+	+	+	+	?	?	?	Yes	Assess equipment availability, expertise availability and legislative/policy constraints
10	+	+	+	+	+	+	+	Not required	
11	+	+	?	+	?	?	?	Yes	Assess patient preference, equipment availability, expertise availability and legislative/policy constraints
12	?	?	?	+	?	?	?	Yes	Assess quality of evidence, strength of recommendation, patient preference, intervention availability, expertise availability and legislative/policy constraints
13	+	?	+	+	+	+	+	Yes	Assess strength of recommendation
Total	**12**	**11**	**11**	**13**	**10**	**10**	**10**	**4**	
%	**92**	**85**	**85**	**100**	**77**	**77**	**77**	**31**	

Note: +, yes item is met; −, no item is not met;? unsure if item is met

**Table 2:** Summary of final panel judgements compared with IWGDF judgements for all IWGDF wound healing recommendations

No	Problem	Desirable effects	Undesirable effects	Quality of evidence	Values	Balance of effects	Acceptability	Applicability/feasibility	Decision	Comment
1	=	=	=	=	=	=	=	=	Adopt	Adopted in screening
2	=	=	=	=	=	=	=	=	Adopt	Adopted in screening
3	=	=	=	=	=	=	=	=	Adopt	Adopted in screening
4	=	=	=	=	=	=	=	=	Adopt	Adopted in screening
5	=	=	=	=	=	=	=	=	Adopt	Adopted in screening
6	=	=	=	=	=	=	=	=	Adopt	Adopted in screening
7	=	=	=	=	=	=	=	=	Adopt	Adopted in screening
8	=	=	=	=	=	=	=	=	Adopt	Adopted in screening
9	+ yes	+ moderate	? small	+ low	+ probably no important uncertainty or variability	+ probably favours the intervention	? don’t know	+ don’t know	Adapt	Adapted to include need for informed consent
10	=	=	=	=	=	=	=	=	Adopt	Adopted in screening
11	+ yes	+ moderate	? small	+ moderate	? probably no important uncertainty or variability	+ probably favours the intervention	+ probably yes	- no	Adapt	Adapted to reflect unavailability of intervention in Australia
12	+ yes	? trivial	? trivial	+ low	? possibly important uncertainty or variability	+ does not favour either intervention or comparison	+ don’t know	+ probably no	Adapt	Adapted strength of recommendation
13	+ yes	+ small	? small	+ low	? probably no important uncertainty or variability	+ does not favour either intervention or comparison	+ probably yes	+ probably yes	Adapt	Adapted strength of recommendation and to include nutrition as part of standard care

Note: +, panel agreed with original IWGDF judgement; −, panel disagreed with original IWGDF judgement;?, panel unsure if agreed with original IWGDF judgement due to lack of IWGDF information on judgement; =, panel agreed with original IWGDF judgements during screening (see Table 1); *QoE* Quality of evidence

**Table 3:** Summary of the original IWGDF recommendation compared with the new Australian guideline recommendations for wound healing

No	Original IWGDF Recommendation	Decision	New Australian Recommendation
1	Remove slough, necrotic tissue, and surrounding callus of a diabetic foot ulcer with sharp debridement in preference to other methods, taking relative contraindications such as pain or severe ischemia into account (GRADE strength of recommendation: strong; quality of evidence: low)	Adopted	As stated in original IWGDF Recommendation
2	Dressings should be selected principally on the basis of exudate control, comfort and cost (strong; low)	Adopted	As stated in original IWGDF Recommendation
3	Do not use dressings/applications containing surface antimicrobial agents with the sole aim of accelerating the healing of an ulcer (strong; low)	Adopted	As stated in original IWGDF Recommendation
4	Consider the use of the sucrose-octasulfate impregnated dressing as an adjunctive treatment, in addition to best standard of care, in noninfected, neuro-ischaemic diabetic foot ulcers that are difficult to heal (weak; moderate)	Adopted	As stated in original IWGDF Recommendation
5	Consider the use of systemic hyperbaric oxygen therapy as an adjunctive treatment in non-healing ischaemic diabetic foot ulcers despite best standard of care (weak; moderate)	Adopted	As stated in original IWGDF Recommendation
6	We suggest not using topical oxygen therapy as a primary or adjunctive intervention in diabetic foot ulcers including those that are difficult to heal (weak; low)	Adopted	As stated in original IWGDF Recommendation
7	Consider the use of negative pressure wound therapy to reduce wound size, in addition to best standard of care, in patients with diabetes and a post-operative (surgical) wound on the foot (weak; low)	Adopted	As stated in original IWGDF Recommendation
8	We suggest not using negative pressure wound therapy in preference to best standard of care in nonsurgical diabetic foot ulcers (weak; low)	Adopted	As stated in original IWGDF Recommendation
9	Consider the use of placental-derived products as an adjunctive treatment, in addition to best standard of care, when the latter alone has failed to reduce the size of the wound (weak; low)	Adapted	Consider the use of placental derived products with informed consent as an adjunctive treatment, in addition to best standard of care, when the latter alone has failed to reduce the size of the wound (weak; low)
10	We suggest not using growth factors, autologous platelet gels, bioengineered skin products, ozone, topical carbon dioxide, and nitric oxide in preference to best standard of care (weak; low)	Adopted	As stated in original IWGDF Recommendation
11	Consider the use of autologous combined leucocyte, platelet and fibrin as an adjunctive treatment, in addition to best standard of care, in noninfected diabetic foot ulcers that are difficult to heal (weak; moderate)	Adapted	Consider the use of autologous combined leucocyte, platelet and fibrin as an adjunctive treatment, in addition to best standard of care, in non-infected diabetic foot ulcers that are difficult to heal only if this adjunctive treatment becomes approved for use in Australia (weak; moderate)
12	Do not use agents reported to have an effect on wound healing through alteration of the physical environment including through the use of electricity, magnetism, ultrasound, and shockwaves in preference to best standard of care (strong; low)	Adapted	We suggest not using agents reported to have an effect on wound healing through alteration of the physical environment including through the use of electricity, magnetism, ultrasound and shockwaves in preference to best standard of care (weak; low)
13	Do not use interventions aimed at correcting the nutritional status (including supplementation of protein, vitamins and trace elements, pharmacotherapy with agents promoting angiogenesis) of patients with a diabetic foot ulcer, with the aim of improving healing, in preference to best standard of care (strong; low)	Adapted	We suggest not using interventions aimed at correcting the nutritional status (including supplementation of protein, vitamins and trace elements, pharmacotherapy with agents promoting angiogenesis) of patients with a diabetic foot ulcer, with the aim of improving healing, but nutritional status should be reviewed and adequate daily nutritional requirements should be met as part of best standard of care. (weak; low).

Note: underlined wording indicates the specific adapted changes to the original IWGDF recommendation

We received four responses (one individual and three organisations) to the public consultation survey with three (75%) responding that they agreed that the guideline should be approved as the new Australian wound healing guideline, that the guideline would be supported by the majority of their colleagues and if approved they would encourage its use in practice. All de-identified feedback comments received during public consultation and the panel’s responses to each comment were collated and are available on the Diabetes Feet Australia website. Based on the collated public consultation feedback, the guideline was revised, approved by the panel and Australian DFD Guidelines working group, and endorsed as the new *Australian guideline on wound healing interventions to enhance healing of foot ulcers* by ten peak national bodies including Wounds Australia, Australian Podiatry Association, Australian and New Zealand Society for Vascular Surgery, Australasian Society for Infectious Diseases, Australian Orthotic Prosthetic Association, Pedorthic Association of Australia, Australian Advanced Practicing Podiatrists - High Risk Foot Group, Australian Aboriginal and Torres Strait Islander Diabetes-related Foot Complications Program, Australian Diabetes Society and Diabetes Feet Australia.

Outlined below are each of the thirteen Australian recommendations for wound healing interventions to enhance healing of DFU. In each section, the panel has detailed the question addressed by the recommendation, the Australian recommendation(s), the panel’s decision on adopting, adapting or excluding the recommendation, and the summarised (and detailed justifications if applicable) for each recommendation. Also included are considerations for implementation of the recommendation in Australia, including for the subgroups of geographically remote and Aboriginal and Torres Strait Islander people. Lastly, we addressed monitoring and potential further priorities for research for each recommendation. A glossary of definitions is available at the end of this paper.

### Question one

In individuals with diabetic foot ulcers, which method of debridement should be used to promote healing?

#### Recommendation 1

Remove slough, necrotic tissue, and surrounding callus of a diabetic foot ulcer with sharp debridement in preference to other methods, taking relative contraindications such as pain or severe ischemia into account (GRADE strength of recommendation: strong; quality of evidence: low).

##### Decision: adopted

Rationale: The panel decided to adopt this recommendation as our judgements agreed with all the IWGDF judgements for this recommendation, including for the strength of recommendation, quality of evidence, patient values, and that sharp debridement is widely acceptable and applicable in the Australian context.

##### Summary justification

The panel agreed with the IWGDF that although the quality of supporting evidence for this recommendation was low, the strength of recommendation was strong for debridement in general as the balance of effects favoured removing slough, necrotic tissue and callus over not removing it for healing of DFU. We also agreed with the IWGDF that there is limited evidence supporting alternative forms of debridement (e.g., larval, ultrasonic, mechanical) as being beneficial to wound healing, and all was at high risk of bias [9]. Furthermore, sharp debridement is now widely considered to be part of best standard of DFU care based on broad international expert opinion that it is superior to other forms of debridement [9, 21] and that the resources and expertise to perform sharp debridement are readily available in Australia and at relatively low cost.

##### Implementation considerations

Debridement is widely acknowledged to be beneficial in removing surface debris, slough and nonviable tissue, which leaves clean and viable tissue, and new bleeding can trigger a new inflammatory phase, and subsequent angiogenesis, formation of granulation tissue, and epithelialisation [21]. The IWGDF Guideline (2019) description of debridement is “the removal of callus or dead tissue that can be surgical (“sharp”) or nonsurgical (e.g., abrasion, chemical)” [9] . However, the panel considered that the definition of sharp debridement still required further clarification for the Australian context. We suggest in this Australian Guideline (2021) that sharp debridement should be defined as “the removal of callus or non-viable tissue that can be surgical (performed in a theatre), or conservative (performed in a clinic), using an aseptic technique with sterilised scalpel or scissors”. Non-sharp debridement techniques may include autolytic, mechanical, larval (bio surgical), ultrasonic, hydro surgical abrasion or chemical methods [25]. We agree with the IWGDF that in the presence of gas-forming infection, abscesses or necrotising fasciitis, that surgical debridement is likely to be urgently required [9].

In Australia, training in sharp debridement of DFU in undergraduate and postgraduate health professional programs is limited. Similarly, formal programs for debridement that are accredited by professional associations, as is present in some countries like the USA are also lacking. Therefore, the panel strongly suggests that sharp debridement should be limited to those who are suitably trained and can demonstrate competence in performing debridement for DFU whilst adhering to local policies and protocols. In addition, that professional peak associations or similar bodies should consider accreditation processes for sharp debridement training courses in vocational wound management programs.

##### Subgroup considerations

Geographical remote people

The panel considers that limited access to healthcare professionals competent in performing sharp debridement in rural and remote communities may exist for some sites. Thus, health professionals in rural and remote communities who lack competence or confidence in performing sharp debridement of DFU should instead establish contacts and referral pathways to health professions competent in debridement in their local areas. The panel similarly encourage health professionals to establish contacts for onsite clinical teaching or consider travelling to metropolitan centers to acquire the necessary knowledge and training to competently perform sharp debridement of DFU.

Aboriginal and Torres Strait Islander people

The panel considers this recommendation is widely applicable to Aboriginal and Torres Strait Islander people if health professionals are trained and competent in sharp debridement, and the procedure of debridement is explained clearly to the recipient.

Other subgroup considerations

The panel agree with the IWGDF that sharp debridement may be contraindicated in individuals with pain or severe ischaemia [9]. Should sharp debridement be clinically contraindicated after comprehensive assessment in these individuals, alternative methods of debridement such as autolytic debridement may be applicable. Considerations for assessment and management of severe peripheral artery disease (PAD) are detailed in the accompanying Australian DFD Guideline on PAD (2021).

##### Monitoring considerations

Regular sharp debridement to remove non-viable or infected tissue is both an important adjunct offloading (from or adjacent to a plantar DFU, where indicated) and wound healing treatment to facilitate DFU healing. Therefore, the panel proposes that organisations monitor the frequency of sharp debridement treatments as a key clinical performance indicator for patients with DFU, and consider benchmarking these treatments to clinical outcomes such as DFU healing rates. Although this is not currently an item in existing national High Risk Foot Service database monitoring systems [26], the panel recommend consideration for inclusion in future iterations as there are likely considerable clinical and health service implications and variations in terms of costs and benefits in time to healing with different debridement regimens.

##### Future research considerations

The panel agrees with the IWGDF Guideline (2019) recommendation that although backed strongly by expert opinion, the quality of evidence for sharp debridement over alternative methods of debridement is low. Therefore, high-quality randomised controlled trials (RCT) are still required for direct comparison of the efficacy of different types, frequency and regimens of debridement to better inform clinical decisions and patient outcomes.

### Question two

In individuals with active diabetic foot ulcers, what is the best dressing/application to choose in addition to usual best care with the aim of enhancing wound healing?

#### Recommendation 2

Dressings should be selected principally on the basis of exudate control, comfort and cost (strong; low)

#### Recommendation 3

Do not use dressings/applications containing surface antimicrobial agents with the sole aim of accelerating the healing of an ulcer (strong; low)

#### Recommendation 4

Consider the use of the sucrose-octasulfate impregnated dressing as an adjunctive treatment, in addition to best standard of care, in noninfected, neuro-ischaemic diabetic foot ulcers that are difficult to heal (weak; moderate).

##### Decision: all three recommendations have been adopted

Rationale

After screening each of these three recommendations which address the original question, the panel agreed with all IWGDF judgements on strength of recommendations, quality of evidence and patient values ratings, plus, that all recommendations were broadly acceptable and applicable in the Australian context.

##### Summary justification for recommendations 2–4

Recommendation 2

The panel agreed with the relevant evidence statement in the IWGDF systematic review that there does not seem to be any evidence to support the balance of effects favouring any wound dressing product over another to achieve ulcer healing across all different types of DFU [21], in addition to the quality of evidence overall being low. Thus, with no dressing product demonstrated to be superior to another for all DFU types, the panel also agreed with IWGDF’s strong strength of recommendation, that dressings should be primarily selected based on the principles of exudate control, comfort and preferably low cost. The panel agreed that such a recommendation based on these principles would be widely acceptable and applicable in the Australian context. Most Australian health services will have the resources and expertise to provide such wound dressings that meet these basic principles for dressing selection. Otherwise, further considerations on how to use this recommendation are provided in the subsequent implementation sub-section below.

Recommendation 3

The panel agreed with the IWGDF that there are studies that seemingly indicate some benefit of individual antimicrobial agents on accelerating DFU healing in locally infected wounds, such as potassium permanganate [27], superoxidised antiseptic solution [28], topical phenytoin [29–31] and herbal extracts. However, all these studies had a high risk of bias based on sub-standard methodology and/or incomplete reporting of data on participant characteristics. Furthermore, the higher quality of these studies mostly reported no difference in DFU healing rates compared to a control of a good (or best) standard of DFU care. Therefore, the quality of supporting evidence is low and current available evidence does not support any additional desirable effect from using antimicrobial dressings over a good standard of DFU care for the primary aim of DFU healing. The panel also agreed with the IWGDF that there are additional undesirable effects of using antimicrobial dressings indiscriminately with most requiring some additional wound healing expertise and expense as compared to a good standard of DFU care. Thus, the balance of effects instead favours using a good standard of DFU care compared with using an antimicrobial dressing, and, supports a strong strength of the recommendation not to use antimicrobial dressings when the primary intention is to accelerate DFU healing.

Recommendation 4

The panel agreed with the IWGDF that firstly, there is at least a moderate quality of supporting evidence for this recommendation. This is based on one high-quality adequately powered RCT demonstrating effectiveness of using a sucrose-octasulfate impregnated dressing over a control dressing to heal DFU that were non-infected, neuro-ischaemic and hard to heal despite best standard of care. However, it is acknowledged that there may be some perception of industry bias in this study due to industry funding of this trial [9]. Thus, the panel agreed that the quality of supporting evidence is moderate until further high quality RCTs support or refute these findings. Secondly, the panel agreed that the balance of effects probably favoured the intervention, based on the one RCT suggesting moderate likely desirable effects on healing based on significantly more DFUs healing at 20 weeks in the intervention group compared to the control group (adjusted odds ratio of 2.6 (95% CI 1.43–4.73)) with a significantly faster mean time to healing; and small likely undesirable effects with quality of life and adverse event outcomes similar in both groups, and cost effectiveness not reported but possibly higher in the intervention group [15]. Subsequently, the strength of this recommendation should remain weak in favour of the sucrose-octasulfate impregnated dressing over control dressings specifically in non-infected neuro-ischaemic DFUs. Lastly, the panel agreed that the intervention is probably acceptable and applicable in the Australian context given increased availability in most services treating DFU, the expertise required to apply these dressings is minimal, and it is approved for use in Australia.

##### Implementation considerations for recommendations 2–4

**Recommendation 2**

In the absence of evidence supporting superiority of one dressing product over another for healing DFUs, dressing selection is paramount as part of a good standard of DFU care. The panel suggests that a good standard of DFU care includes a comprehensive DFU assessment, appropriate debridement, appropriate wound dressings, antimicrobial management if infected, revascularisation considerations if ischaemic and the best available offloading device provided for the patient [14, 32] (see recommendations for other treatments in those accompanying Australian DFD Guidelines) [33–36].

Regarding the wound dressing aspect of this good standard of DFU care, the short-term goals to principally address exudate levels, comfort and cost, along with the overall goals of care and preferences of the patient should be considered when deciding on which wound dressing to select. For example, for a patient with a low exuding non-infected neuropathic DFU, an inexpensive, less absorbent dressing may suffice. Conversely, for a heavily exuding neuropathic DFU, super absorbent foams or polymer dressings may be preferred as they would readily absorb higher amounts of exudate, are comfortable and whilst slightly more expensive, may be more cost-effective, which relates to the dressing efficacy on DFU healing along with the costs of frequency of dressing change. Dressings should be changed when soiled or when strikethrough is observed, and not placed on wounds for periods exceeding manufacturer’s recommendations for use. The ability of the dressing to conform comfortably to the anatomical location is also a high priority. The panel additionally suggests clinicians consider the TIME principle of chronic wound bed preparation and treatment in selecting an appropriate dressing [37].

**Recommendation 3**

Please refer to Recommendation 2 for similar implementation considerations, as well as the panel’s definition of a good standard of wound care for DFU.

**Recommendation 4**

The panel notes that the study population for this recommendation is limited to those with non-infected, neuro-ischaemic DFU, that have not healed after receiving a good standard of DFU care, with ischaemic defined as having moderate PAD (see accompanying Australian DFD Guideline for PAD for definitions) [15, 34]. The panel suggests that before considering using sucrose-octasulfate impregnated dressings, health professionals should confirm the person has moderate PAD and that they have a non-healing DFU after receiving the aforementioned principles of a good standard of DFU care. Non-healing DFUs can be defined as those DFUs that have not reduced in size by > 50% after receiving 4–6 weeks of the above good standard of care. Once these parameters are confirmed the use of sucrose-octasulfate impregnated dressings should be considered in addition to a good standard of DFU care. Please also refer to Recommendation 2 for similar implementation considerations, as well as the panel’s definition of good standard of wound care for DFU in the below glossary of terms section.

##### Subgroup considerations for recommendations 2–4

Geographical remote people

There are no additional considerations for geographical remote people than for those mentioned above.

Aboriginal and Torres Strait Islander people

There are no additional considerations for Aboriginal and Torres Strait Islander people than for those mentioned above.

Other subgroup considerations

Recommendations 2 and 3 are generic recommendations and there are no specific subgroups that will find this unacceptable or are contraindicated. The panel also suggests that there are no contraindications to implementing Recommendation 4 either, however we do note that benefit of this sucrose-octasulfate impregnated dressing has only been demonstrated in those with neuro-ischaemic DFU and benefit in other DFU types is unknown.

##### Monitoring considerations for recommendations 2–4

The panel suggests that along with maintaining a good standard of DFU care, dressings should be monitored and changed when soiled or when strikethrough is observed, and not placed on wounds for periods exceeding the manufacturer’s recommendations for use. Otherwise, the panel encourage the inclusion and capture of different dressing types in High Risk Foot Service database monitoring systems [26] to benchmark the different dressing types’ efficacy on local DFU healing.

##### Future research considerations for recommendations 2–4

The panel agreed with the IWGDF that further high quality RCTs, in accordance with IWGDF standards for conducting and reporting DFU trials, on the clinical utility and effectiveness of most non-antimicrobial and antimicrobial dressings compared with a good standard of DFU care are needed. In addition, that further high quality RCTs on the use of sucrose-octasulfate impregnated dressings are also still required to confirm or refute the existing high quality trial. However, it is noted post publication of the IWGDF Guideline (2019), a further study reported the cost effectiveness of the sucrose-octasulfate impregnated dressing compared with a simple dressing as €6017.25 versus €9928.49 respectively, for direct treatment costs incurred over 20 weeks with complete wound closure as a primary endpoint, indicating that sucrose-octasulfate impregnated dressings may also be cost-effective [38]. It is reasserted future studies are required to confirm this finding.

### Question three

In individuals with active diabetic foot ulcers, does systemic hyperbaric oxygen or topical oxygen therapy in comparison to standard care help promote healing?

#### Recommendation 5

Consider the use of systemic hyperbaric oxygen therapy as an adjunctive treatment in non-healing ischaemic diabetic foot ulcers despite best standard of care (weak; moderate).

##### Decision: adopted

Rationale

The panel decided to adopt this recommendation as our judgements agreed with all the IWGDF judgements for each recommendation, including the strength of recommendation, quality of evidence, patient values and that systemic hyperbaric oxygen therapy (HBOT) is generally applicable and acceptable in the Australian context.

##### Summary justification

The panel agreed with the IWGDF there is a moderate quality of supporting evidence for the use of HBOT on non-healing ischaemic ulcers. This was based on two older RCTs with low risk of bias that showed that in some ischaemic DFUs, HBOT significantly improved healing within 12 months [39, 40]. Seven subsequent heterogenous RCTs with mostly high risk of bias, investigating different DFU types, populations, healing outcomes and follow-up times showed mostly no difference between HBOT and control treatments [21]. Therefore, there is likely some small-to-moderate desirable effects for using HBOT in ischaemic DFU types. It was further agreed that although HBO therapies are available and approved for use in Australia, the cost-effectiveness, accessibility, and acceptability of use in Australia may be limited and because of this, small undesirable effects are likely for using HBOT over good standard of DFU care. Overall, the panel agreed that the balance of effects probably favours HBOT over a good standard of care in moderately ischaemic DFU types only.

##### Implementation considerations

Systemic HBOT aims to overcome wound hypoxia, promote epithelialisation, and subsequently accelerate healing. There is a dose-response relationship for HBOT which often requires patients to attend four to five treatments per week. The total number of treatment sessions varies depending on the response of the wound to HBOT, but often exceeds 6 weeks of treatment in total [41]. Due to the need for frequent, and lengthy treatment sessions, the desirable effects (small-to-moderate benefit on wound healing) and undesirable effects (small risks from increased costs, treatment time and potential adverse effects) should be carefully discussed with the patient, in addition to the requirement to commit to regular attendance for required treatment periods, which may limit a patients’ autonomy. Moreover, the cost-effectiveness of systemic HBOT is yet to be established, although HBOT is subsidized by the Australian Medicare Benefits Schedule for DFU.

The panel notes the study population for this recommendation is limited to those with non-healing, ischaemic DFU that have not healed after receiving a good standard of DFU care, with ischaemic defined as having moderate PAD (see accompanying Australian DFD Guideline for PAD for definitions) [15, 34]. The panel suggests that before considering using systemic HBOT that health professionals should confirm the person does have PAD, has adequate vessel patency and that they have a non-healing DFU after receiving a good standard of DFU care. Please also refer to Recommendation 2 for similar implementation considerations, as well as the panel’s definition of non-healing DFU and a good standard of wound care for DFU.

Where systemic HBOT is considered as an adjunctive mode of treatment in addition to a good standard of care, we recommend referral and comprehensive assessment by the receiving HBOT unit prior to commencement of therapy. The selection of patients for HBOT should include a comprehensive medical assessment and take into consideration patient preferences and overall goals of care. The panel also reiterate that this recommendation is specific to people with ischaemic DFU as there is, as yet, no clear evidence of benefit for HBOT in healing for other DFU types. Furthermore, when applied, systemic HBOT is to be an adjunctive mode of treatment in addition to good standard of care, not as a replacement for such care.

##### Subgroup considerations

Geographical remote people

At the time of writing, there were twelve hyperbaric oxygen units across Australia, all of which were in major metropolitan or major regional areas. Due to the requirement for numerous sessions of daily treatment over several weeks, people living in rural and remote Australia will likely have limited access to this treatment modality, as requirements of travel or the costs of temporary accommodation in close proximity to hyperbaric units may be prohibitive. The panel suggests that in these circumstances, healthcare providers in rural and remote Australia should carefully discuss with their patient the additional travel costs for geographically remote patients along with the small-to-moderate benefit that HBOT treatment is likely to provide for patients with ischaemic DFU. Health professionals who have patients who make an informed decision to undergo HBOT should establish contacts and referral pathways to facilitate access to this treatment modality.

Aboriginal and Torres Strait Islander people

This recommendation is potentially applicable to Aboriginal and Torres Strait Islander people. However, the panel acknowledges that many Aboriginal and Torres Strait Islander people live in geographically remote areas with limited accessibility to HBOT as stated above. The panel also recommends that Aboriginal health workers should be involved where possible to carefully discuss the risks and benefits of HBOT for ischaemic DFU with Aboriginal and Torres Strait Islander people to ensure that patients are able to make a culturally appropriate informed decision.

Other subgroup considerations

Numerous systemic HBOT units in Australia also require patients to be within a confined space for a few hours at a time and a patient’s disposition to claustrophobia should be considered when recommending HBOT.

##### Monitoring considerations

The panel also proposes that organisations include the use of systemic HBOT alongside other wound healing interventions (such as the above wound dressings) in their organisational database monitoring systems to monitor the impact of these wound healing interventions on clinical outcomes and DFU healing rates. Although this is not currently an item in existing national High Risk Foot Service database monitoring systems [26], the panel propose HBOT be considered for inclusion as an intervention worthy of future monitoring.

##### Future research considerations

Since publication of the IWGDF Guidelines in 2019, a systematic review and meta-analysis of HBOT in DFU has shown reduction in major amputation rates, but not wound healing rates, in patients with DFU and PAD [41]. However, the panel agree with the IWGDF that further appropriately powered high quality RCTs are required to confirm the (cost)-effectiveness of HBOT [21]. Further, the type of DFU that would benefit most from HBOT, and duration of therapy is still unknown with further high-quality research in this area still needed [21].

#### Recommendation 6

We suggest not using topical oxygen therapy as a primary or adjunctive intervention in diabetic foot ulcers including those that are difficult to heal (weak; low).

##### Decision: adopted

Rationale

The panel decided to adopt the recommendation as our judgements agreed with all the IWGDF judgements for the recommendation, including the strength of recommendation, quality of evidence, patient values and that topical oxygen therapy is generally applicable and acceptable in the Australian context.

##### Summary justification

The panel agrees with the IWGDF regarding the lack of evidence supporting the balance of effects favouring topical oxygen therapy over a good standard of DFU care to achieve wound healing. Although a few published studies showed some beneficial effect of topical oxygen in wound healing, most were at high risk of bias due to methodological flaws [9], and although two high quality RCTs [42, 43] with low risk of bias have been published, they have conflicting results. Moreover, topical oxygen therapy is likely to require additional wound healing expertise to use and be more expensive than a good standard of DFU care. Thus, the balance of effects probably favours a good standard DFU care over topical oxygen therapy. The panel agreed with the IWGDF for not recommending topical oxygen therapy as a primary or adjunctive treatment in DFU healing until more high quality RCTs are available and the true effect of topical oxygen therapy on wound healing is ascertained [9].

##### Implementation, subgroup and monitoring considerations

Topical oxygen therapy supplies continuous or cyclical diffusion of oxygen over the surface of the wound. As this recommendation favours a good standard of care over the intervention in DFU healing, there are no additional considerations for implementation, monitoring and subgroups than those outlined for a good standard of care in earlier wound dressing recommendations (See Recommendations 2–4).

##### Future research considerations

The panel agree with the IWGDF that further high-quality, well conducted RCTs compared with a good standard of DFU care controls are required to substantiate any future desirable effects over undesirable effects before topical oxygen therapy can be recommended for use in people with DFU.

### Question four

In individuals with active diabetic foot ulcers, does negative pressure wound therapy (NPWT) in comparison to standard care help promote healing? If so, when? And which setting?

#### Recommendation 7

Consider the use of negative pressure wound therapy (NPWT) to reduce wound size, in addition to best standard of care, in patients with diabetes and a post-operative (surgical) wound on the foot (weak; low).

#### Recommendation 8

We suggest not using negative pressure wound therapy in preference to best standard of care in nonsurgical diabetic foot ulcers (weak; low).

##### Decision: both recommendations 7 and 8 were adopted

Rationale

The panel decided to adopt these recommendations as our judgements agreed with all the IWGDF judgements for these recommendations, including the strength of recommendation, quality of evidence, patient values, and that NPWT is widely available and acceptable in the Australian context.

##### Summary justification for recommendations 7–8

**Recommendation 7**

The panel agreed with the IWGDF that whilst there were four studies showing shortened time to healing in those using NPWT when used as an adjunctive treatment for DFU that have undergone surgical intervention, these were all at high risk of bias and had numerous methodological flaws [9]. Thus, the balance of effects slightly favours the use of NPWT in addition to best standard of care for patients with a surgically-treated DFU, although the quality of supporting evidence is low. Furthermore, NPWT is applicable and acceptable, and currently widely used within the Australian context, although the cost-benefit of its use has not been demonstrated [9].

**Recommendation 8**

The panel agree with the IWGDF that some studies reporting on the use of NPWT on non-surgically treated DFU showed some statistically significant benefit compared to standard care. However, those studies were at high risk of bias with incomplete reporting, statistical analysis and other significant methodological flaws [9]. Thus, the desirable effects are likely to be small-to-moderate at most and the undesirable effects of additional time, cost and expertise are also small-to-moderate. Overall, the balance of effects does not favour NPWT over a good standard of care to enhance healing of non-surgically treated DFU. It was further agreed that the strength of recommendation suggesting not to use NPWT in people with non-surgical treated DFU in preference to best standard of care is weak, and the quality of evidence is low [9].

##### Implementation considerations for recommendations 7–8

**Recommendation 7**

Negative pressure wound therapy (NPWT) is the application of an intermittent or continuous subatmospheric pressure to the system foam or gauze filler, which more recently has been shown to actually provide a positive pressure to the surface of a wound and facilitates the draining of wound exudate, the promotion of angiogenesis, granulation tissue and assists with wound contraction [44]. Apart from appropriate patient selection, there are no specific considerations for implementation.

**Recommendation 8**

As this recommendation favours standard care over the intervention, there are no considerations for implementation.

##### Subgroup considerations for recommendations 7–8

Geographical remote people

Surgical procedures on DFU and the use of NPWT thereafter are likely to be instituted in a large tertiary or regional hospital or community settings, which may not be in close proximity to people living in geographically remote areas. Continuation of NPWT upon discharge also relies upon the availability of competent staff as well as access to NPWT equipment, which again may be unavailable in geographically remote areas. When implementing NPWT in surgically treated DFUs in people from geographically remote areas, health professionals should consider the access to NPWT equipment and availability of competent staff to facilitate NPWT treatment. The panel also recommends discussion of the potential desirable (potentially faster wound healing) and undesirable effects (higher costs or a longer admission) with patients so an informed decision can be made. Staff in geographically remote areas may also consider undertaking training to overcome any skill gap, including in provision of post-hospital discharge NPWT with portable devices or NPWT single use dressings.

Aboriginal and Torres Strait Islander people

In a similar manner to considerations for people in geographically remote locations, the panel also considers that Aboriginal and Torres Strait Islander people who reside in rural and remote Australia may have to travel away from their families to receive surgical care for DFU. Hence, considerations for initiating NPWT in surgically treated DFUs should take into consideration availability of staff and equipment in the patient’s community upon discharge and the desirable and undesirable effects discussed as part of informed consent and patient-centered care.

Other subgroup considerations.

The panel is unaware of any subgroups for which NPWT would be unacceptable.

##### Monitoring considerations for recommendations 7–8

**Recommendation 7**

The panel propose organisations monitor the use and effect of NPWT on healing surgical wounds in organisational database monitoring systems for patients with DFU. Although this is not currently an item in existing national High Risk Foot Service database monitoring systems [26], it is proposed for inclusion in the future.

**Recommendation 8**

As this recommendation favours a good standard of DFU care over the intervention (NPWT in people with chronic, non-surgical DFU), there are no monitoring considerations needed for this recommendation.

##### Future research considerations for recommendations 7–8

The panel agrees with the IWGDF that robust RCTs are required to determine the (cost)-effectiveness of NPWT as an adjunctive treatment in addition to best standard of care for surgical and non-surgically treated DFUs [21]. The panel is cognisant that NPWT is occasionally used in non-surgical DFUs in Australia and recommend that similar high quality RCTs evaluating its (cost) effectiveness be conducted to support its use in Australia.

### Question five

In individuals with active diabetic foot ulcers that are hard-to-heal, does the use of placental derived products in addition to standard care in comparison to standard care alone help promote healing?

#### Recommendation 9

Consider the use of placental derived products with informed consent as an adjunctive treatment, in addition to best standard of care, when the latter alone has failed to reduce the size of the wound (weak; low).

##### Decision: adapted

Rationale

The panel decided to adapt the original IWGDF recommendation, based on having a differing judgement to the IWGDF for the acceptability rating. Therefore, we re-worded the IWGDF recommendation to include the need to specifically obtain informed consent prior to using placental-derived products to enhance DFU healing.

##### Summary justification

The panel agreed with the IWGDF overall on the balance of effects and low quality of evidence. The key area of disagreement was around acceptability; noting that the product had only very recently been approved in Australia (December 2020) and thus, the panel felt that placental derived products may not be widely acceptable, and particularly for people identifying with certain religious faiths. The recommendation was adapted to overtly emphasise the need for fully informed consent being provided by the patient to use a placental derived product after careful explanation as to the nature of the product. Otherwise, the panel agreed with the IWGDF that feasibility of wider use of this intervention is also unknown.

##### Detailed justifications

After full assessment of all IWGDF evidence [9, 21] and any additional Australian evidence or expert opinion, the panel had similar judgements to the IWGDF for most EtD criteria that proved justification for the original IWGDF recommendation. Our judgements were:

Problem a priority: Yes. The panel agreed with the IWGDF that DFU are a significant health problem in Australia [5, 6] and internationally [1, 2].

Desirable effects: Moderate additional desirable effects. The panel agreed with the IWGDF that overall, desirable effects of the use of placental derived products are likely to be moderate as several studies showed benefit of its use on healing (improvement in time to healing [45–47] and incidence of healing [48]) when used in conjunction with standard care as compared to standard care alone.

Undesirable effects: Small additional undesirable effects. The IWGDF did not report on any undesirable effects in either the IWGDF Guideline (2019) or the systematic review. However, the panel rated this as small, based on our expert opinion that this is a non-invasive topical therapy with unknown adverse events, patient satisfaction, and cost, with the cost likely to be higher for these products than good standard of care.

Quality (or certainty) of evidence: Low. The panel agreed with the IWGDF that the quality of evidence was low due to the only eligible studies having moderate to high risk of bias.

Values of outcomes: Probably no important uncertainty. Although the IWGDF did not specifically report on the values of outcomes relating to the use of placental derived products, our expert opinion was there is probably no important uncertainty or variability on how patients value the main outcomes of healing.

Balance of effects: Probably favours the intervention. The panel considered the balance of effects probably favours placental derived products compared to best standard of care, based on the difference between moderate desirable effects (benefits) and small undesirable effects (risks).

Acceptability: Unknown. The IWGDF did not report on any issues relating to acceptability. However, at the time of writing, placental derived products were very new to the Australian context (approved for use in December 2020) and thus the skills, equipment and cost requirements, along with acceptability of its use were unknown. The panel acknowledges (see subgroup considerations) that this product may also be unacceptable to certain faith groups. Furthermore, there may be a potential or actual lack of clinical expertise to sustain use of these therapies nationally.

Feasibility: Unknown. The panel agreed with the IWGDF that the feasibility of placental derived products in wound care is unknown.

##### Implementation considerations

Placental membranes are those that contain a host of growth factors, extracellular matrix, fibroblasts and epithelial cells that are conducive to wound healing. These products are available in cryopreserved preparations containing live cells and growth factors, as well as dehydrated products containing growth factors only [21]. The IWGDF collectively analyzed the effectiveness of these interventions over standard care, but whilst there was evidence of limited benefit, the evidence is insufficient to support the superiority of one (type of) product over another. To our knowledge, only the dehydrated version of placental derived products is now available, but not yet widely used within Australia, having not long received approval with the Therapeutic Goods Administration (TGA); whilst cryopreserved and live preparation options are not yet available. Therefore, due to the lack of knowledge of this new product in Australia, we suggest that health professionals considering using placental derived products adhere to the manufacturer’s recommendations for use. Availability of placental membrane derived products are also limited by availability of suitable donors, potentially contributing to the final product cost.

##### Subgroup considerations

Geographical remote people

The equipment and expertise likely to be required for using placental derived products may be significant and people living in rural and regional Australia may have difficulties in accessing it.

Aboriginal and Torres Strait Islander people

In addition to all above considerations, the panel suggest this intervention may be unacceptable to some Aboriginal and Torres Strait Islander people due to traditional beliefs causing reticence in using placental derived products.

Other subgroup considerations

The use of placental derived products may be unacceptable to people who identify with certain religious backgrounds; for this reason, the panel have highlighted the importance of obtaining specific full informed consent for using this product before using it as an intervention.

##### Monitoring considerations

In addition to general monitoring considerations outlined for other dressings in earlier recommendations (see Recommendations 2–4), to the panel’s knowledge there were no specific monitoring implications for this recommendation. However, the panel again strongly suggest that any health professionals using this product strictly adhere to manufacturer’s recommendations for safe use and monitoring.

##### Future research considerations

Current evidence supporting the use of placental derived products in wound care is limited. There is insufficient evidence to support superiority of one type of placental derived product over another. Data are also lacking on adverse events such as risk of infection, health economic outcomes and applicability in daily practice [21]. The panel agree with the IWGDF that high quality RCTs for efficacy and cost-effectiveness of these interventions are essential to strengthen the rationale for their use [9].

### Question six

In individuals with active diabetic foot ulcers that are hard to heal, do products designed to improve ulcer healing by altering the biology: growth factors, platelet related products, bioengineered skin products and gases or a combination of leucocyte platelet and fibrin, in comparison to standard care alone help promote healing?

#### Recommendation 10

We suggest not using growth factors, autologous platelet gels, bioengineered skin products, ozone, topical carbon dioxide, and nitric oxide in preference to best standard of care (weak; low).

##### Decision: adopted

Rationale

The panel decided to adopt these recommendations as our judgements agreed with all the IWGDF judgements for these recommendations, including the strength of recommendation, quality of evidence, patient values, and that these products were not widely available and acceptable in the Australian context.

##### Summary justification

The panel agreed with the IWGDF that although there is some evidence supporting the use of products that alter the biology (growth factors and platelet related products) in promoting wound healing, they are at high risk of bias and have severe methodological flaws [21]. Moreover, the costs and expertise involved in using these interventions may be significant, and thus the balance of effects probably favours a good standard DFU care over their use and has not been recommended. The panel agreed with the IWGDF providing a weak strength of recommendation for not recommending the use of products that alter the biology of wounds in promoting wound healing until more high quality RCTs become available, the true effect of these interventions on wound healing is ascertained, and its acceptability and applicability to the Australian context are known.

##### Implementation, monitoring and subgroup considerations

As this recommendation favours a good standard care over the intervention in treating DFU, there are no additional considerations for implementation, monitoring and subgroups than those outlined for a good standard of care in earlier wound dressing recommendations (See Recommendations 2–4).

##### Future research considerations

These intervention(s) require further high-quality, well conducted RCTs compared with a good standard of care controls to substantiate any future desirable effects over undesirable effects before they can be recommended for use in people with DFU.

#### Recommendation 11

Consider the use of autologous combined leucocyte, platelet and fibrin as an adjunctive treatment, in addition to best standard of care, in non-infected diabetic foot ulcers that are difficult to heal only if this adjunctive treatment becomes approved for use in Australia (weak; moderate).

##### Decision: adapted

Rationale

The panel adapted the recommendation, based on a differing judgement to the IWGDF for the feasibility rating as to our knowledge this product has not been approved for use in Australia. Therefore, the panel re-worded the IWGDF recommendation to include the need for this product to be approved in Australia before it can be used.

##### Summary justification

Whilst the panel disagreed with the IWGDF on feasibility we agreed on all other GRADE EtD criteria (see detailed justifications below). The evidence behind the use of autologous combined leucocyte, platelet and fibrin as an adjunctive treatment is based on one high quality RCT reporting statistically significant improved primary (wound healing) and secondary (time to healing) outcomes compared to standard care, with no significant adverse events or effects associated with its use [16]. Thus, the balance of effects probably favours the intervention with the quality of evidence rated as moderate and strength of recommendation weak. Whilst there are restrictions for its use in specific populations, the panel considered its use would be probably be acceptable for most patients in Australia. However, to our knowledge, this product is not currently approved for use in Australia hence precluding its use in Australia. In recognition of the moderate quality of favourable evidence supporting this product compared to a good standard of care alone in certain patients, the panel have adapted this recommendation to enable the product to potentially be used once it becomes approved and available for use in Australia.

##### Detailed justifications

After full assessment, the panel had mostly similar judgements to IWGDF on justifications for this recommendation [9, 21], with the exception being feasibility.

Problem a priority: Yes. The panel agreed with the IWGDF that the problem of DFU is significant in Australia [5, 6] and globally [1, 2].

Desirable effects: Moderate additional desirable effects. There was one high quality RCT [49] reporting on the use of autologous combined leucocytes, platelets and fibrin in patients with hard to heal ulcers. The panel considered the likely desirable effect size was moderate based on a 12% significant difference in ulcer healing favouring the intervention compared to a control of a best standard of care [21]. Furthermore, time to healing as a secondary outcome was significantly shorter in the intervention group (*p* = 0.034, 16). Overall, the panel rated the desirable effects as moderate, in agreement with the IWGDF.

Undesirable effects: Small additional undesirable effects. The IWGDF’s systematic review failed to report the details of secondary outcomes or serious adverse events that were cited in the original RCT. However, the RCT reported that there were likely trivial effects for secondary outcomes as key findings reported for infection and anaemia rates were not significantly different between both arms in the reported RCT [16]. However, there were no findings reported on costs and in our expert opinion the costs of this product are likely to be much higher than best standard of care, plus, it was unclear if the requirement for patients to attend weekly for preparation and application of the autologous combined leucocytes, platelet and fibrin may be undesirable and difficult to achieve. Thus, the panel rated the undesirable effects as small.

Quality (or certainty) of evidence: Moderate. The panel agreed with the IWGDF that the quality of evidence is moderate, based on the one RCT of high quality, but was currently lacking cost-effectiveness data and has not had findings supported by a further RCT as yet. Thus, the quality of evidence was rated as moderate [9].

Value of outcomes: Probably no important uncertainty. The panel agreed with the IWGDF valuing wound healing as the main critical outcome.

Balance of effects: Probably favours the intervention. The panel considered the balance of effects probably favours the use of autologous combined leucocytes, platelet and fibrin with best standard of care compared to best standard of care alone, based on the difference between moderate desirable (benefits) and small undesirable effects (risks) in patients with hard to heal ulcers [9].

Acceptability: Probably yes. The panel agreed with the IWGDF that the use of autologous combined leucocytes, platelet and fibrin would probably be acceptable to most patients and providers in healthcare settings that typically provide such treatment. However, the panel also acknowledged that it is currently not available for use in Australia (not approved by the TGA) and as such costs of implementing this product are unknown. There would, however, be subgroups for which this would be unacceptable, as described further below.

Feasibility: Not feasible. The panel agreed with the IWGDF that the autologous combined leucocytes, platelet and fibrin requires weekly visits for preparation and application which may have significant cost implications. However, at the time of publication the most significant barrier to feasibility is until TGA approved the product is not available for use in Australia.

##### Implementation considerations

At the time of publication, the autologous combined leucocyte, platelet and fibrin as an adjunctive treatment was not available in Australia. However, should the Australian TGA approve its use the panel suggests health professionals consider the following before using it:

1. Skills in phlebotomy are required. The autologous combined leucocyte, platelet and fibrin intervention is obtained by first drawing 18 mL of a patient’s venous blood, which is subsequently spun for 20 minutes according in a pre-specified program in a centrifuge, producing a final three layered patch that can be applied to the foot ulcer using an aseptic technique [16]. It is then covered by a low adherent, knitted viscose rayon primary dressing and a protective secondary dressing.
2. It is unclear whether specialist equipment such as the centrifuge will need to be purchased; even if existing pathology laboratory centrifuges can be used, these are not commonly co-located with multidisciplinary DFU units and may present a significant challenge for implementation.

##### Subgroup considerations

Geographical remote people

Sites in rural and remote Australia may sometimes lack health professionals with skills in phlebotomy, or equipment necessary to perform this intervention.

Aboriginal and Torres Strait Islander people

This intervention may be unacceptable to Aboriginal and Torres Strait Islander people as traditional beliefs may cause reticence in using blood-related products.

Other subgroup considerations

Small subgroups of the population may refuse blood transfusion based on faith and cultural beliefs. One key example is those of Jehovah’s Witness faith, who deem autologous predonation of blood products unacceptable [50]; approximately 0.4% of the Australian population identify with this faith [51]. These preferences should be taken into consideration in assessing suitability of a person for this intervention. The panel also highlights that the RCT excluded patients with interdigital ulcers due to difficulties in measuring and placing the patch on the wound [16] and thus this dressing may be impractical for these wound types.

##### Monitoring considerations

In addition to general monitoring considerations outlined for other dressings in earlier recommendations (see Recommendations 2–4), to the panel’s knowledge there were no specific monitoring implications for this recommendation, however, it is strongly advised to strictly adhere to manufacturer’s recommendations for safe use and monitoring.

##### Future research considerations

The panel agreed with the IWGDF that future high quality RCTs and cost-effectiveness analyses should be undertaken to help support or refute the findings of the one high-quality RCT on this intervention to date.

### Question seven

In individuals with active diabetic foot ulcers that are difficult to heal, does the use of other products that alter wound biology through mechanical and physical means (lasers, shockwaves, ultrasound, magnetism, and electric current) in addition to standard care in comparison to standard care alone help promote healing?

#### Recommendation 12

We suggest not using agents reported to have an effect on wound healing through alteration of the physical environment including through the use of electricity, magnetism, ultrasound and shockwaves in preference to best standard of care (weak; low).

##### Decision: adapted

Rationale

The panel have adapted the recommendation, based on having differing judgements to the IWGDF for undesirable effects, balance of effects and feasibility ratings. Therefore, the panel downgraded the strength of the recommendation from “strong” to “weak” and changed the wording accordingly to reflect this conditional/weak recommendation.

##### Summary justification

The panel disagreed with the IWGDF that the magnitude of undesirable effects (such as adverse events, patient dissatisfaction) from these interventions that alter wound biology through mechanical and physical means (lasers, shockwaves, ultrasound, magnetism, and electric current) outweighed the desirable effects (see detailed judgements below) to such an extent that the panel could be certain that the balance of effects favours the best standard of care control over the intervention(s). Instead, the panel found the balance of effects likely does not favour either these intervention(s) or best standard of care as they both produce similar desirable and undesirable effects, resulting in the recommendation being downgraded. The panel, however, agreed it was not known if the intervention(s) would be acceptable for most patients and providers of DFU care in Australia and that the intervention(s) was probably not feasible to implement widely across Australia due to differences in TGA approval for these therapies, costs and expertise needed for use. Overall, the panel’s ratings suggested a weak/conditional recommendation against using the intervention compared to best standard of care.

##### Detailed justifications

After full assessment, the panel had similar judgements to IWGDF on justifications for this recommendation, except for undesirable effects and feasibility:

Problem a priority: Yes. The panel agreed that the problem of DFU is significant in Australia [5, 6] and globally [1, 2].

Desirable effects: Trivial additional desirable effects. The panel agreed with the IWGDF that there did not seem to be an additional desirable effect for the intervention(s) compared with the control on healing [9]. This was based on numerous studies in this topic being of low quality/high risk of bias investigating a range of heterogenous interventions compared with a range of heterogenous standard of care controls showing little or no significant benefit on healing [21]. The panel rated desirable effects as trivial.

Undesirable effects: Trivial additional undesirable effects. The IWGDF Guideline (2019) or systematic review did not address any undesirable effects of the intervention(s), but did indicate at least a small-to-moderate undesirable effect as they concluded a balance of effects strongly favouring the control. However, the panel rated undesirable effects as being trivial, based on our expert opinion, that these are generally non-invasive topical therapies, although acknowledging that there is a lack of evidence on adverse events, cost effectiveness or patient dissatisfaction.

Quality (or certainty) of evidence: Low. The panel agreed with the IWGDF that the quality of evidence is low, based on the numerous aforementioned low-quality, heterogenous studies reporting inconsistent findings.

Value of outcomes: Possibly important uncertainty. The IWGDF reported on the value of wound healing as the most critical outcome. Whilst the panel did not necessarily disagree, it was our expert opinion that other outcomes such as pain and frequency of treatment may be critical and valued outcomes to patients and therefore rated the possibility of important uncertainty or variability on the value of outcomes.

Balance of effects: Does not favour either the interventions or control, based on the difference between our ratings of trivial desirable and trivial undesirable effects.

Acceptability: Unknown. The panel were uncertain if most patients and providers would find these treatments acceptable based on the uncertain evidence for benefits and risks and that the interventions included in this recommendation are very heterogenous in terms of likely patient satisfaction, costs, treatment and frequency. Thus, the panel did not know if these collective interventions would be acceptable to most patients or not.

Feasiblity: Probably no. The panel rated the collective intervention as probably not feasible to use due to differences in their TGA approval status for use in Australia, costs, and necessary expertise to be able to use widely in Australia.

##### Implementation, subgroup and monitoring considerations

As this recommendation favours a good standard of care over the intervention(s) in treating DFU, there are no additional considerations for implementation, subgroup or monitoring than those outlined for a good standard of care in earlier wound dressing recommendations (See Recommendations 2–4).

##### Future research considerations

These intervention(s) require further high-quality, well conducted RCTs compared with a good standard of care controls to substantiate any future desirable effects over undesirable effects before they can be recommended for use in people with DFU.

### Question eight

In individuals with active diabetic foot ulcers that are difficult to heal, do interventions aimed at correcting the nutritional status (including supplementation of vitamins and trace elements, pharmacotherapy with agents promoting angiogenesis) in comparison to standard care help promote healing?

#### Recommendation 13

We suggest not using interventions aimed at correcting the nutritional status (including supplementation of protein, vitamins and trace elements, pharmacotherapy with agents promoting angiogenesis) of patients with a diabetic foot ulcer, with the aim of improving healing, but note that nutritional status should be reviewed, and adequate daily nutritional requirements should be met as part of best standard of care (weak; low).

##### Decision: adapted

Rationale

The panel adapted this recommendation, based on having differing judgements to the IWGDF for undesirable effects and balance of effects ratings, and the need to ensure there was no confusion on ensuring that appropriate nutritional intake for general health and wellbeing is maintained and not compromised by confusion around the wording of this recommendation. Therefore, the strength of the recommendation was downgraded from “strong” to “weak” and added the qualifying phrase “nutritional status should be reviewed, and adequate daily nutritional requirements should be met as part of best standard of care” to ensure that such a recommendation was not misinterpreted.

##### Summary justification

The panel disagreed with the IWGDF that the balance of effects favoured best standard of care alone over nutritional supplementation in combination with best standard of care to heal people with DFU. The panel considered the balance of effects to favour neither the intervention nor control. However, the panel also considered that adequate daily nutritional intake is essential for wound healing, collagen synthesis and angiogenesis in other wound types such as pressure injuries [52]. The panel acknowledge that although DFU and pressure injuries have different aetiologies, there are similarities in principles of wound closure for both types of chronic wounds. The panel have clarified in the recommendation that patients with DFU should have their nutritional status reviewed to ensure daily nutritional requirements are met as part of best standard of general health care and wound care.

##### Detailed justifications

After full assessment, the panel had similar judgements to IWGDF on justifications for this recommendation, except for undesirable effects and feasibility:

Problem a priority: Yes. The panel agreed with the IWGDF that the problem is significant in Australia and globally.

Desirable effects: Small additional desirable effects. The panel agreed with the IWGDF that desirable effects of nutritional supplementation are small. Although numerous studies [53, 54] reported statistically significant benefit from nutritional supplementation on secondary outcomes of reducing ulcer size, there were major methodological flaws and inconsistencies ranging from poor definition of best standard of care, uncertainty around compliance and high risk of bias. Conversely, two high quality RCTs reported no differences between a nutritional supplementation intervention and best standard of care control on the primary outcome of healing at a certain time point, whilst another reported apparent improvement. All studies were on different nutritional supplementation interventions [21]. Overall, the panel concluded that there was likely a small desirable effect.

Undesirable effects: Small additional undesirable effects. Unfortunately, the IWGDF Guideline (2019) or systematic review did not report the undesirable effects of adverse events, patient dissatisfaction or costs from nutritional supplementation in the aforementioned numerous trials. However, the panel’s expert opinion deemed undesirable effects to be small and mostly relating to a potentially large additional ongoing cost of nutritional supplements which may be a burden.

Quality (or certainty) of evidence: Low. The panel agreed with the IWGDF that the quality of evidence is low, based on the numerous aforementioned trials with heterogenous quality, interventions, controls, and outcome definitions reporting inconsistent findings.

Values of outcomes: Probably no important uncertainty. The IWGDF did not report on any values apart from wound healing.

Balance of effects: Does not favour the intervention or control, based on the difference between our ratings of small desirable and small undesirable effects.

Acceptability: Probably yes. The IWGDF Guideline (2019) or systematic review did not address acceptability of nutritional supplements to patients and providers. However, in our expert opinion the panel considered nutritional supplements are probably acceptable in most patients considering the wide-spread use of nutritional supplements in the general Australian population.

Feasibility: Probably yes. The IWGDF Guideline (2019) or systematic review did not address feasibility or applicability of nutritional supplements to enhance wound healing. However, the panel rated the intervention as being probably feasible to implement for most patients, although it was recognized that the cost of such nutritional supplements without strong evidence of added benefit can pose a significant financial burden on patients and that the fresh food required to meet basic nutritional (dietary) needs may be limited in rural and remote communities as described below.

##### Implementation considerations

The panel felt there were no specific implementation implications for this recommendation, however, advised consideration be again given to the general implementation implications outlined for a good standard of DFU care in earlier wound dressing recommendations (See Recommendations 2–4), plus, the need for nutritional screening to determine if daily nutritional intake for general health and wellbeing is adequate or not when implementing this recommendation. The panel refers readers to the Australian Dietary Guideline on recommendations on the types and amounts of foods Australians should consume to meet nutritional requirements [55], as well as nutritional support recommendations for specific subgroups of people with diabetes and diabetes-related complications [56]. The panel additionally suggests, where indicated, referral to a dietitian experienced in diabetes management for additional nutritional support should be considered.

##### Subgroup considerations

Geographical remote people

People living in rural and remote regions of Australia may have reduced access to fresh food such as fruit and vegetables required for adequate daily nutritional intake. The panel acknowledges inequitable access to fresh food may have implications for wound healing. Attempts should be made to rectify poor access to fresh food where possible.

Aboriginal and Torres Strait Islander people

The recommendation is acceptable to Aboriginal and Torres Strait Islander people. However the panel acknowledges that like people living in rural and remote regions of Australia, there are multiple access challenges to affordable fresh produce within many Australian and Torres Strait Island communities.

Other subgroup considerations

The panel were not aware of any other subgroups where this recommendation would be unacceptable, however, refers readers to the Australian Dietary Guideline on recommendations on the types and amounts of foods Australians should consume to meet nutritional requirements or any potential contraindications [55].

##### Monitoring considerations

The panel propose there were no specific monitoring implications for this recommendation, however, advise consideration of the general monitoring implications in this guideline when implementing this recommendation.

##### Future research considerations

These nutritional supplement intervention(s) require further high-quality, well conducted RCTs compared with a good standard of care controls to substantiate any future desirable effects over undesirable effects before they can be specifically recommended for use in wound healing in people with DFU.

## Discussion

### Recommendations summary

For the first time in 10 years, new Australian wound healing intervention guidelines to heal DFUs have been developed. The panel systematically evaluated the 13 recent IWGDF Guideline (2019) evidence-based wound healing intervention recommendations to potentially adopt or adapt to the Australian context. Following this process, nine recommendations were adopted and four were adapted for the Australian context. The main reasons for adapting recommendations were: specifying the need for informed consent (recommendation 9), unavailability of the intervention in Australia (recommendation 11), re-wording to clarify best standard of care (recommendation 13), and downgrading the strength of two recommendations (recommendations 12 and 13). Overall, five wound healing interventions have been identified as having the potential to improve wound healing in specific DFU types when implemented in conjunction with other best standard of DFU care recommended in accompanying Australian DFD guidelines. These were the sucrose-octasulfate impregnated dressing, systemic hyperbaric oxygen therapy (HBOT), negative pressure wound therapy (NPWT), placental-derived products, and the autologous combined leucocyte, platelet and fibrin dressing. Of these five interventions, three (sucrose-octasulfate impregnated dressing, systemic HBOT and NPWT) are approved and available in Australia, one (placental-derived products) is newly registered with the ARTG and the other (autologous combined leucocyte, platelet and fibrin dressing) is not yet approved for use in Australia.

### Differences to previous guidelines

There are thirteen recommendations in this new Australian 2021 Wound Healing Guidelines compared with seven recommendations made in the previous 2011 Australian DFD Guidelines relating to wound healing interventions. The similar recommendations that remain in this new guideline are that: local sharp debridement of DFU be performed if not contraindicated (Recommendation 1); wound dressings should be selected based primarily on exudate control, comfort or cost as there is a lack of evidence for one wound dressing product being more effective than others to heal all different DFU types (Recommendation 2); and that there is some evidence for the use of systemic HBOT for ischaemic DFU (Recommendation 5) and NPWT for post-surgical DFU (Recommendation 7) [10].

However, there were multiple new recommendations made in this new guideline potentially reflecting the new high-quality evidence gained in this field over the last decade [9]. These new recommendations included a number of new wound product interventions that are appropriate to use and other wound products that are not appropriate to use for certain DFU types. Those new wound products to consider using included: sucrose-octasulfate impregnated dressings for hard-to-heal neuro-ischaemic DFU (Recommendation 4), placental derived products (Recommendation 9) and an autologous combined leucocyte, platelet and fibrin dressing for hard-to-heal DFU (Recommendation 11); although the latter products have only very recently been approved for use in Australia or not yet approved, respectively. Those wound products not recommended over a best standard of DFU wound healing care intervention (i.e. Recommendation 1 and 2) included: antimicrobial dressings (Recommendation 3); topical oxygen therapy (Recommendation 6); NPWT for non-surgically treated DFU (Recommendation 8); growth factors, autologous platelet gels, bioengineered skin products, ozone, topical carbon dioxide, and nitric oxide (Recommendation 9); electricity, magnetism, ultrasound and shockwaves therapy (Recommendation 12); and nutritional supplements (Recommendation 13).

Interestingly, two recommendations made in the previous 2011 Guideline were not addressed in this new 2021 Guideline: Larval therapy and Skin replacement therapies (cultured skin equivalents and skin grafting) were recommended to be considered in specialist centres in the previous guideline. This difference may be attributed to the difference in methodology and research questions used by the IWGDF in 2019 and the NHMRC in 2011. Thus, due to the methodology used in this guideline to adapt existing guidelines, we are unable to judge the appropriateness of these previous guideline recommendations and can only encourage health professionals to independently review and evaluate current evidence on the use of these therapies. Otherwise we suggest questions on larval therapy and skin replacement therapies should be included in future Australian DFD guidelines if possible.

### Implementation considerations summary

**Fig. 1:**
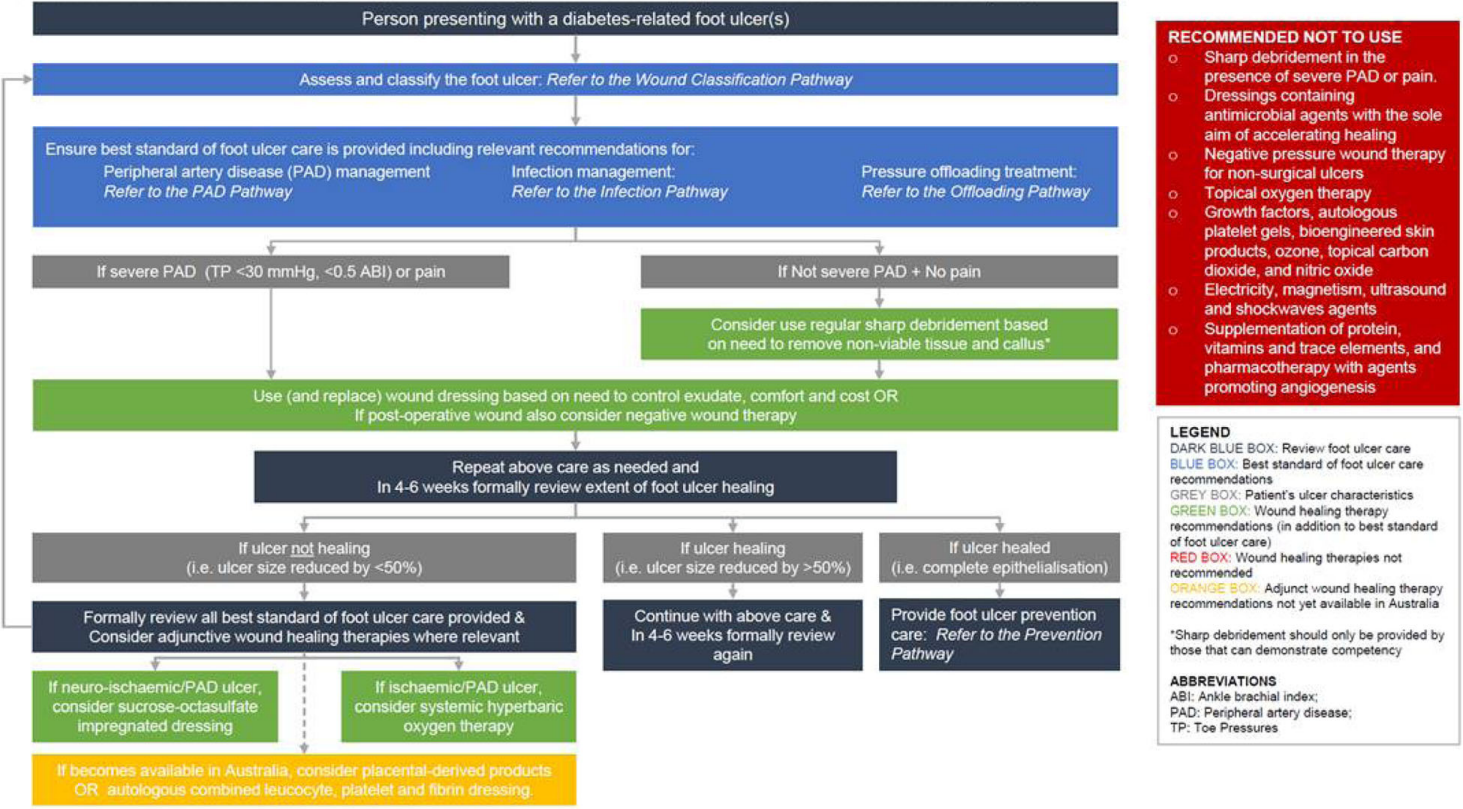
Australian evidence-based clinical pathway on wound healing interventions for people with diabetes-related foot ulcers (DFU)

All recommendations in this new 2021 Australian guideline should be implemented in conjunction with a good standard of DFU care. The panel defines this as including recommendations made on comprehensive DFU assessment (see wound classification guideline), appropriate debridement (Recommendation 1), wound dressings (Recommendation 2), antimicrobial management if infected (see infection guideline), revascularisation considerations if ischaemic (see PAD guideline) and the best available offloading device (see offloading guideline) [14, 32] in the accompanying Australian DFD Guidelines [33–36] (Fig. 1).

The panel acknowledge that the interventions recommended in this guideline in addition to good standard of care are likely to be more expensive (to both the individual and healthcare system), and cost-effectiveness data is generally lacking. In selecting or considering a recommended intervention to promote DFU healing, the health professional should carefully discuss with the patient their overall goals of care, short-term outcomes desired for any wound dressing of exudate control, comfort and cost, and the desirable (improved wound healing), and undesirable effects (any adverse events, increased consultations needed and costs) of any suggested wound product recommended in the process of obtaining informed consent.

### Subgroup considerations summary

Overall, all thirteen recommendations are widely applicable to Aboriginal and Torres Strait Islander people. The panel acknowledge that Aboriginal and Torres Strait Islander populations residing in non-metropolitan areas may encounter difficulties in accessing good standard care, as well as certain wound healing interventions recommended in this Australian DFU Guideline. In such instances, the panel strongly recommend health professionals working in these communities develop required skillsets or referral pathways to ensure Aboriginal and Torres Strait Islander people with DFU can access equitable care. The panel have also highlighted that recommendations 9 and 11, consisting of placental and blood-related products, may not be acceptable to Aboriginal and Torres Strait Islander people and specific consent should be obtained prior to using these interventions. The main considerations for geographical remote populations relate to accessibility of specific wound healing strategies as identified within stated recommendations. Where local accessibility, or lack of skilled staff may present key limitations, as in the case of systemic HBOT or NPWT therapies respectively, the panel recommends developing referral pathways or clinical training to acquire competency. Other key subgroup considerations generally relate to contraindications of certain wound healing interventions, for example pain and severe ischaemia for sharp debridement, or patient preferences based on religious beliefs (blood and placental derived products). Thus, the panel has highlighted the need to obtain informed consent when these interventions are implemented.

### Monitoring considerations summary

The panel recommend the use of existing High Risk Foot Service database monitoring systems [26] for monitoring the use and effectiveness of wound healing interventions on healing their patients with DFU. Further, it is acknowledged that important therapy regimen option details of several wound healing interventions such as frequency of debridement, NPWT or systemic HBOT treatments that are not currently collected in this database should be considered for inclusion in the future, as part of real-world data.

### Future research considerations summary

The panel agree with the IWGDF that it is imperative that all 2016 IWGDF key reporting standards for DFU studies are adhered to [14]. This should further enhance the body of evidence, strength of recommendation and quality of evidence behind all wound healing interventions to enhance the fields’ understanding of what works best to promote DFU healing. The lack of (cost-)effectiveness investigated for numerous therapies were also a consistent theme across all 13 wound healing recommendations, and future research should implement cost-effectiveness analyses in their trials to shed important new light on these interventions for patients and providers [14].

### Strengths and limitations

There are a number of strengths in the development of this Guideline. Firstly, the Guideline Working Development Group followed NHMRC-recommended ADAPTE and GRADE procedures to identify suitable international source guidelines [20, 22] and to systematically adapt the IWGDF Guideline (2019) to the Australian context. An independent panel of multi-disciplinary experts in the care of people with DFU were involved in this process. Moreover, the panel included a consumer representative and an Aboriginal and Torres Strait Islander expert who aided decision making, which the original IWGDF Guideline lacked.

A limitation of this guideline, however, was the reliance on the IWGDF’s research questions and accompanying systematic reviews in identifying all relevant evidence for the panel to review, meaning evidence published after the 2019 review may have been missed. However, the panel were able to include and review any additional Australian literature of which they were aware. Finally, the panel acknowledge some opinions and views may have been missed, although a widely representative international expert panel were consulted in all decision-making processes, and the guidelines were widely available during a public call for consultation from all Australian health professionals, researchers and peak bodies.

## Conclusion

The original IWGDF Guideline on use of interventions to enhance healing of chronic foot ulcers in diabetes (2019) recommended five wound healing interventions that have the potential to enhance wound healing in DFU when used in conjunction with good standard of care [9, 21]. In these new Australian guidelines on wound healing interventions to enhance healing of foot ulcers: Part of the 2021 Australian evidence-based guidelines for diabetes-related foot disease, the IWGDF Guidelines (2019) have been systematically adapted for the Australian context, in particular geographically remote and Aboriginal and Torres Strait Islander people. The guideline also highlights important considerations for implementation and monitoring, as well as future research priorities for Australia. In implementing and monitoring these recommendations, as well as the other recommendations in accompanying chapters of the new Australian DFD guidelines, it is anticipated patients with DFU should experience better care, and subsequently improved healing outcomes, reducing the national burden and inequalities amongst all Australians with or at risk of DFU.

## Supplementary Information

**Additional file 1.**

## Data Availability

Data sharing is not applicable to this article as no datasets were generated or analysed during the current study.

## Abbreviations

DFU: Diabetes-related foot disease
EBR: Evidence-Based Recommendations
EtD: Evidence to Decision
GRADE: Grading of Recommendations, Assessment, Development and Evaluations
HBOT: Hyperbaric Oxygen Therapy
IWGDF: International Working Group of the Diabetic Foot
NHMRC: National Health and Medical Research Council
NPWT: Negative Pressure Wound Therapy
PAD: Peripheral Artery Disease
RCT: Randomised Controlled Trial
TGA: Therapeutic Goods Administration
