# Supplementary material for: Australian guideline on wound healing interventions to enhance healing of foot ulcers: part of the 2021 Australian evidence-based guidelines for diabetes-related foot disease
Source: J Foot Ankle Res. 2022 May 25;15:40. doi: 10.1186/s13047-022-00544-5 (PMC9131573; doi:10.1186/s13047-022-00544-5)
Supplement: Supplementary file 1 — Additional file 1. [file 13047_2022_544_MOESM1_ESM.docx]

Professor Catherine Bowen
Editor-in-Chief,

*Journal of Foot and Ankle Research*

Dr Professor Bowen

Re: JFAR-D-21-00181 “**Australian guideline on wound healing interventions to enhance healing of foot ulcers: Part of the 2021 Australian evidence-based guidelines for diabetes-related foot disease**”

May we thank you and the reviewers for kindly considering our manuscript for publication in your journal. We appreciate the constructive reviews, and after making revisions in response to each comment, we believe this has further strengthened the manuscript.

We have carefully considered and responded to each of the reviewers’ individual comments below and where possible have made corresponding revisions accordingly. Please see our point-by-point response to reviewers’ comments, along with the extracts of all revisions made, with page and line numbers referred to in the attached revised manuscript.

In addition to the amendments as recommended by the authors and editorial team, two authors have revised affiliations since the submission of the manuscript in November 2021. These revisions are detailed on page 1.

Again, we thank you and the reviewers for your constructive comments, and we believe our manuscript has only benefited from these comments. Please do not hesitate to contact us if you have any further queries and we look forward to your reply.

Your sincerely,

Pam Chen

On behalf of all authors.

Prof Kerlyn Carville, Dr Terry Swanson, A/Professor Peter A Lazzarini, A/Professor James Charles, Ms Jane Cheney, Dr Jenny Prentice, Ms Nytasha Purcell, Professor Stephen M Twigg, on behalf of the Australian Diabetes-related Foot Disease Guidelines & Pathways Project, Diabetes Feet Australia.

**COMMENTS TO THE AUTHORS:**

**Editorial comments:**

***Please add the following:***

1. ***Add a line in the abstract at the end of the results as follows: The six new guidelines and the full protocol can be found at:*** [***https://www.diabetesfeetaustralia.org/new-guidelines/***](https://www.diabetesfeetaustralia.org/new-guidelines/)

【Response】We thank the editor for their comments and have made this addition to the manuscript *(page 4, lines 69-70 in marked-up manuscript).*

2. ***Add the following statement to the declarations section: “The authors also note that this manuscript has been published online in full and can be found on the Diabetes Feet Australia website:*** [***https://www.diabetesfeetaustralia.org/new-guidelines/***](https://www.diabetesfeetaustralia.org/new-guidelines/)***. Furthermore, the authors confirm they have been fully authorised by Diabetes Feet Australia, the copyright owners of these 2021 Australian evidence-based guidelines for diabetes-related foot disease, to submit this manuscript for publication in the Journal of Foot and Ankle Research.”***

【Response】We thank the editor for their comment and have made this addition to the manuscript *(page 63, lines 1363-1368 in marked-up manuscript).*

**Reviewer: One**

1. ***The article shows a comprehensive and methodical approach in adopting international recommendations to a national level, with justified adaptation to national and local demographics***

【Response】We thank the reviewer for their time in reviewing the manuscript and providing complimentary comments.

**Reviewer: Two**

1. ***This is an important piece of work. Although it relates to Australian guidelines, the processes described in assessing and applying international consensus guidelines, will be immensely useful to other nations or areas considering such an undertaking. It is well written, although very long. This may put some readers off, but you have included detailed methodology that would allow for replication of the processes you have described. To shorten the paper would lose the detail of how the recommendations were assessed and validated for the Australian context.***

【Response】We thank the reviewer for their time in reviewing the manuscript and for providing complimentary and constructive comments which will enhance the manuscript.

2. ***In the first paragraph of ‘Background’ I would have liked to know the prevalence of diabetes in Australia to provide some additional context. Within that, what is the prevalence of DFUs?***

【Response】We thank the reviewer for their comment and have included prevalence data for both diabetes, as well as diabetes-related foot ulceration in people with diabetes in the background section of the manuscript (*page 5, lines 87-88)*

3. ***Otherwise, I have no significant amendments to suggest and commend the authors for their work***

【Response】We thank the reviewer again for their kind comments.
